# Competing Bifurcations Determine Symmetry Breaking During Droplet Snaps on Smooth Patterned Surfaces

**DOI:** 10.1021/acs.langmuir.4c02908

**Published:** 2024-11-06

**Authors:** Lucile Bisquert, Élfego Ruiz-Gutiérrez, Marc Pradas, Rodrigo Ledesma-Aguilar

**Affiliations:** † Institute for Multiscale Thermofluids, School of Engineering, University of Edinburgh, The King’s Buildings Mayfield Road, Edinburgh EH9 3FB, United Kingdom; ‡ School of Engineering, Newcastle University, Claremont Road, Newcastle upon Tyne NE1 7RU, United Kingdom; § School of Mathematics and Statistics, The Open University, Milton Keynes, MK7 6AA, United Kingdom

## Abstract

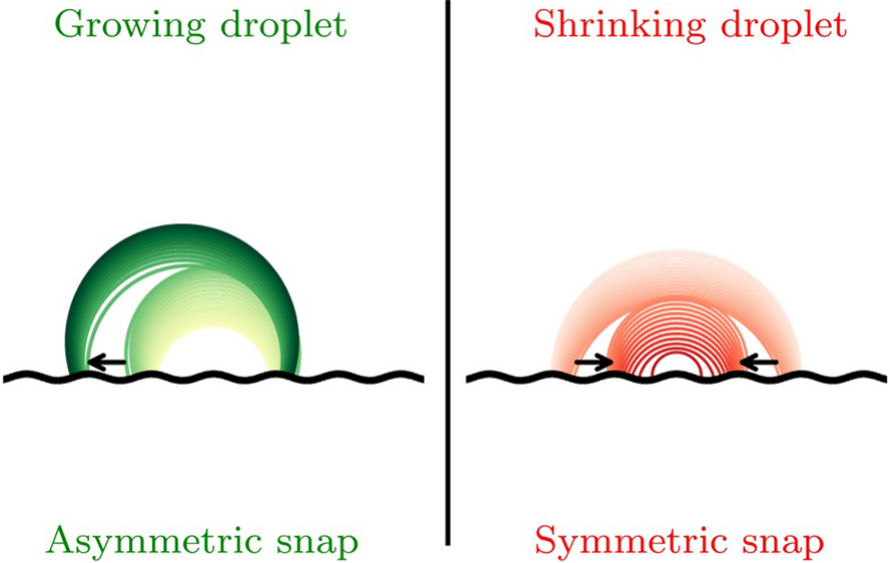

The shape and stability of a droplet in contact with a solid surface is affected by the chemical composition and topography of the solid, and crucially, by the droplet’s size. During a variation in size, most often observed during evaporation, droplets on smooth patterned surfaces can undergo sudden shape and position changes. Such changes, called snaps, are prompted by the surface pattern and arise from fold and pitchfork bifurcations which respectively cause symmetric and asymmetric motions. Yet, which type of snap is likely to be observed is an open fundamental question that has relevance in the rational design of surfaces for managing droplets. Here we show that the likelihood of observing symmetric or asymmetric snaps depends on the distance between fold and pitchfork bifurcation points and on how this distance varies for droplets that grow or shrink in size on surfaces patterned with a smooth topography. Our results can help develop strategies to control droplets by exploiting smooth surface patterns but also have broader relevance in situations where different types of bifurcations compete in determining the stability of a system, for instance in snap-through instabilities observed in elastic media.

## I Introduction

The interaction of droplets with structured solid surfaces is important in practical applications, including printing, heat transfer and fog harvesting.^1−3^ Many of these applications also involve changes in the size of the droplets. For example, when a droplet interacts with a porous solid surface, as occurs in printing, the liquid can either be absorbed or expelled by the pores of the surface. Similarly, in drying and fog harvesting, a droplet undergoes phase changes, and these come with a variation of the droplet size.

Droplet evaporation provides a familiar illustration of how droplets interact with a solid surface while undergoing a slow reduction in size. On a perfectly smooth and flat surface, an evaporating droplet can shrink keeping a constant shape. This situation is known as the constant-contact-angle mode of evaporation, since the droplet keeps a constant apparent contact angle as its size varies.^4−6^ Microscopic surface roughness causes pinning of the contact line, which results in a constant contact-area mode of evaporation where the apparent contact angle decreases as the droplet evaporates.^4^ In practice, one often observes a combination of these two modes, which is known as the stick–slip mode,^7^ or a mode where the contact line suddenly depins from small surface features, known as the stick-jump mode.^8,9^

A different mode, called snap evaporation,^10,11^ occurs when a droplet interacts with a surface equipped with a smooth chemical or topographical pattern whose typical length scale is comparable to the droplet size. Snaps correspond to sudden motions of the edges of the drop as it evaporates. Unlike the jumps observed in the stick-jump mode, snaps are not depinning events caused by sharp corners or strong chemical defects. Instead, they arise due to fold and pitchfork bifurcations of the droplet shape triggered by a change in the droplet size. Each bifurcation type leads to different kinds of snap. Fold bifurcations result in symmetric snaps, where the edges of the droplet move simultaneously, keeping the plane of symmetry. Pitchfork bifurcations give rise to asymmetric snaps, where only one edge of the droplet moves, thus leading to a break in the symmetry and a repositioning of the droplet on the solid pattern.

Snaps are likely to occur in any situation involving a change in size of a droplet in contact with a smooth patterned solid. Such situations can involve a size increase or decrease driven by flow, or a phase change, such as dropwise condensation. Therefore, an important question is how the underlying structure of the fold and pitchfork bifurcations determines which type of snap arises as a droplet varies in size, and how the competition between symmetric and asymmetric snaps changes depending on whether the droplet grows or shrinks, or on the wettability of the underlying surface. Such knowledge can help better rationalize the design of surfaces that exploit snap behavior to control the shape and position of droplets on solid surfaces.

In this paper we study the competition of symmetric and asymmetric snaps of droplets undergoing an increase or a decrease in size on smooth patterned surfaces of different shape and equilibrium contact angle. We use the lattice–Boltzmann numerical simulation scheme reported in ref^10^ to model the time-evolution of both growing and shrinking droplets in contact with a sinusoidal surface topography of controlled variation in the contact angle. Our simulation results show that, under the same conditions, symmetric snaps are favored for shrinking droplets, while asymmetric snaps dominate for growing droplets. To understand this effect, we introduce a model based on the Young–Laplace equation, which we use to study the stability of the droplet around equilibrium states. The stability analysis reveals that the separation between fold and pitchfork bifurcation points differs depending on whether the droplet grows or shrinks. This difference results in a higher likelihood of asymmetric snaps for growing droplets vs shrinking droplets. We also study the effect of the amplitude of the pattern and the average contact angle of the droplet on the surface, finding that asymmetric snaps are generally more likely on surfaces with larger pattern amplitude and higher equilibrium contact angle.

The rest of the paper is organized as follows. In Section II we present the formulation of the diffuse-interface model (Section II.A) and the sharp-interface model (Section II.B). In Section III we present the results of this work. Finally, in Section IV we present our conclusions.

## II Methodology

In this section we present the mathematical methods used to study the effect of a variation in size on the stability of a droplet in contact with a smooth periodic solid surface. We first discuss a diffuse-interface model which we use to study a slow variation in the size of the droplet driven by diffusive mass exchange with the surrounding gas phase. We then present a sharp-interface model of the shape of the droplet that we use to study the stability of the droplet upon a lateral displacement of its equilibrium position on the surface.

### II.A Diffuse-Interface Model

#### II.A.1 Governing Equations

**Figure 1:**
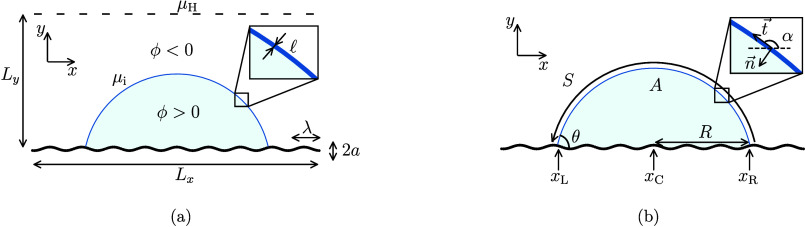
Schematic representation of a two-dimensional droplet in contact with a smooth sinusoidal solid surface. (a) Diffuse interface approximation. A single droplet undergoes a variation in size driven by diffusive mass exchange with the surrounding gas phase. The droplet and the surrounding phase are defined by a phase field, ϕ, which varies smoothly between the droplet (ϕ > 0) to the surrounding phase (ϕ < 0) across an interfacial region of thickness . Diffusion is driven by a difference between the chemical potential at the top boundary, μ_H_, and at the droplet’s interface, μ_i_. The topography of the solid surface is defined by its amplitude, *a*, and wavelength, λ. (b) Equilibrium configuration of a droplet in the sharp-interface approximation. The shape of the interface is determined by the Young–Laplace equation and the Young–Dupré condition. The configuration of the droplet is characterized by the base radius, *R*; cross-sectional area, *A*; arc length, *S*; contact angle, θ; and the positions of its edges, *x*_L_ and *x*_R_, and center, *x*_C_. The local orientation of the interface is described by the unit tangent and normal vectors, t⃗ and n⃗, and the orientation angle relative to the horizontal, α.

We consider a two-dimensional binary-fluid model that allows the coexistence of two immiscible phases that represent the liquid droplet and the surrounding gas phase, as shown in Figure 1a. This approach has been validated previously to study the snaps in the context of droplet evaporation in three dimensions.^10^ The binary fluid occupies a region of size Ω and is bounded on one side by a solid surface ∂Ω. The composition of the phases is modeled using a phase field, , which varies smoothly across a diffuse interfacial region. The Helmholtz free energy of the system is^12,13^
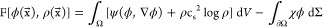
1The first integral in eq 1 corresponds to the bulk free energy, where the free-energy density is defined as
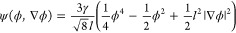
2Here, the polynomial corresponds to a double-well potential that allows the formation of the two equilibrium fluid phases, whose bulk equilibrium values are ϕ = ±1. In the following, the liquid droplet corresponds to ϕ > 0, while the surrounding gas phase corresponds to ϕ < 0. The square-gradient term penalizes the transition from one phase to the other, allowing the formation of a diffuse interface, where  represents the interface thickness and γ the surface tension. The second term in the volume integral of eq 1 corresponds to an ideal-gas contribution, where ρ is the mass density and  is the speed of sound in the fluid. The second integral in eq 1 represents the interaction between the fluid and the solid boundary, where the constant χ controls the wettability of the solid surface. For example, letting χ > 0 leads to a decrease of the free energy if the solid is in contact with the droplet (ϕ > 0), thus favoring that the solid is wet by the liquid.

We are interested in a slow continuous variation of the droplet size, which we achieve through diffusive mass transfer between the droplet and the surrounding phase. Let us introduce the chemical potential field
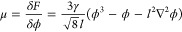
3In equilibrium, the chemical potential satisfies μ = const. Out of equilibrium, a difference in chemical potential creates a diffusive flux,
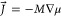
4where *M* is a mobility. Imposing conservation of ϕ then leads to the Cahn–Hilliard equation
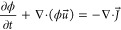
5where *u⃗* is the velocity field of the fluid. The Cahn–Hilliard equation can be used to model a variation of the droplet’s mass by imposing an imbalance in the chemical potential between the droplet’s surface (where μ ≈ 0) and the surrounding phase. Here, we impose this difference by setting the chemical potential at the top boundary of the system to an out-of-equilibrium value, μ_H_ (see Figure 1a). For example, setting μ_H_ > 0 results in a net diffusive mass transport toward the droplet, leading to its growth. Conversely, setting μ_H_ < 0 results in a shrinking droplet.

To complete the governing equations of the model we write the mass and momentum conservation equations, which correspond to the continuity and Navier–Stokes equations:
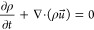
6and
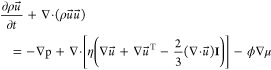
7where *p* = ρ*c*_s_^2^ is the pressure and η is the dynamic viscosity. In eq 7, **I** corresponds to the identity tensor and the superscript T indicates a transpose operation.

#### II.A.2 Boundary Conditions

To solve eqs 5–7 we need to specify boundary conditions at the solid surface, as well as at the open boundaries on the top, right and left edges of the domain.

For the solid surface, we consider a sinusoidal profile of local height
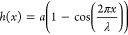
8where *a* is the amplitude and λ the wavelength. The surface-energy term in eq 1 leads to a boundary condition for the phase field:
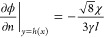
9where *n* is the coordinate normal to the solid surface. It can be shown that the parameter χ is related to the equilibrium contact angle, θ, by^14^
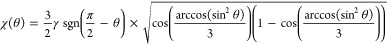
10where sgn is the sign function. To ensure that no transport occurs across the solid surface, we impose a no-flux condition for the chemical potential
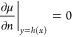
11a no-slip boundary and impenetrability condition for the velocity,
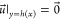
12and a local mass conservation condition,
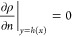
13The top of the domain is treated as an open boundary. As discussed above, we introduce a difference in chemical potential between the droplet surface and the boundary to drive a variation of the droplet size. We set the value of the chemical potential at the boundary, μ_H_, according to eq 3 in which we assume that the chemical potential is relaxed to a steady state and thus, . Therefore, the boundary conditions are
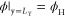
14and
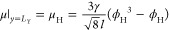
15The density at the top boundary is set as
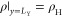
16which helps maintain a uniform pressure away from the droplet. The boundary condition for the velocity is
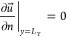
17which allows free mass flux across the boundary.

For the left and right edges of the domain, we apply periodic boundary conditions, i.e.,
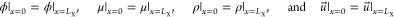
18

#### II.A.3 Simulation Setup and Integration Algorithm

We use a rectangular simulation domain of dimensions (*L*_X_, *L*_Y_) = (128, 128) in lattice units. The wavelength of the sinusoidal surface is set to λ = *L*_X_/9 and the amplitude is varied in the range 0.018λ ≤ *a* ≤ 0.088λ. These values guarantee the presence of both fold and pitchfork bifurcation points,^11^ and the observation of multiple snaps during the variation of the droplet size. To introduce the effect of surface heterogeneity, we allow small fluctuations of the local contact angle along the surface around an average value. Here we use a Gaussian distribution with an average in the range 45° ≤ θ ≤ 120° and a standard deviation 0° ≤ σ_θ_ ≤ 5°. The rest of the parameters are set as follows (all in simulation units if not specified otherwise). The interface thickness is set to  = 1.4, resulting in a Cahn number *Cn* ≡ /*L*_X_ = 0.01. The mobility is fixed to *M* = 18 and the surface tension to γ = 1 × 10^–4^. To ensure a smooth transition of the viscosity between the two phases, we use the truncated linear interpolation
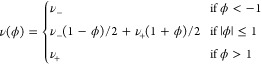
19between two bulk values, ν_+_ = 0.083 in the droplet and ν_–_ = 0.0083 in the surrounding gas phase.

We consider two types of simulation configurations. The first corresponds to a droplet of initial base radius *R* = 0.1*L*_X_ that is allowed to grow by setting ϕ_H_ = −0.7 at the top boundary. The second is a droplet with *R* = 0.45*L*_X_ that shrinks by setting ϕ_H_ = −1.3. For both configurations, the initial lateral position of the droplet is set at the center of the domain, *x*_C_ = 0.5*L*_X_. The initial velocity field and density fields are fixed to  and . In the following, we report all quantities in units of the wavelength of the pattern λ and the characteristic time scale of diffusive mass transport, *T* = *L*_Y_^2^/*M*μ_H_.

To integrate the equations we use a free-energy lattice–Boltzmann algorithm.^13^ Details of the numerical method are provided in the Supporting Information.

### II.B Sharp-Interface Model

The diffuse interface approach detailed in Section II.A allows the study of droplets undergoing snaps by means of numerical simulations. To rationalize the origin of the snaps, we use a sharp-interface model to study the stability of the droplet upon a small lateral displacement from a reference thermodynamic equilibrium state, and how the stability is affected as the size of the droplet varies. In mechanical equilibrium the shape of the droplet is governed by the Young–Laplace equation,
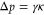
20where Δ*p* is the pressure jump across the interface and κ is the interface curvature.

**Figure 2:**
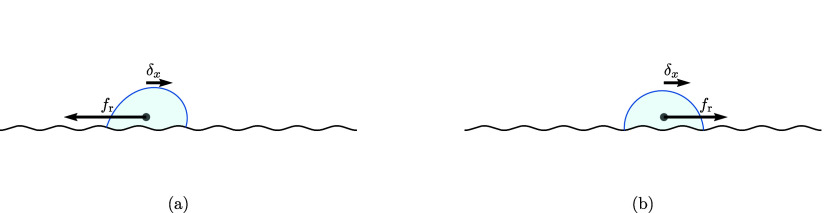
Stable and unstable configurations on a sinusoidal surface. The droplet is displaced by δ_*x*_ from its equilibrium position on a peak or a valley of the topography. (a) Stable configuration: the response force, *f*_r_, is opposite to the direction of the displacement. (b) Unstable configuration: δ_*x*_ and *f*_r_ point in the same direction.

Consider a lateral displacement δ_*x*_ which shifts the droplet from a reference equilibrium position causing a distortion to the interface shape. This distortion will cause a response capillary force, *f*_r_, which will either point in the direction of the displacement or against it. If both the displacement and the capillary force point in the same direction the configuration is unstable. If they point in opposite directions the configuration is stable. To determine the capillary force, we impose a condition of mechanical equilibrium where the capillary force is balanced by a uniform external force. For a given displacement from thermodynamic equilibrium our aim is to use eq 20 to compute the interface shape and the corresponding response capillary force, which in turn determines the stability of the droplet. Figure 2 shows examples of the distortion of the interface shape created by a lateral displacement. In Figure 2a the response capillary force *f*_r_ points in the opposite direction to δ_*x*_, thus indicating a stable droplet configuration. In contrast, for the configuration shown in Figure 2b, δ_*x*_ and *f*_r_ point in the same direction, indicating an unstable configuration.

To solve eq 20, we first introduce the parametrization of the interface shape
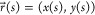
21where *r⃗* is the position vector of a point on the interface and *s* is the arc length (see Figure 1b). Therefore, the tangent vector to the interface is
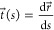
22We introduce the orientation angle relative to the *x*-axis, α = α(*s*). Therefore, the tangent vector reads
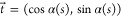
23In order to compute the curvature from the tangent vector, we use the Frenet–Serret identity
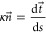
24where *n⃗* = (−sin α(*s*),cos α(*s*)) is the vector normal to the interface. It follows from eq 24 that the curvature is given by κ = dα/d*s*.

Let us consider the effect of a lateral displacement on the position of the droplet. The displacement changes the position of its center, *x*_C_, defined as the midpoint between the left and right edges. For simplicity, we assume that the corresponding distortion of the interface is caused by a uniform force density acting in the *x* direction, ρ*g*_*x*_. Therefore, the pressure profile obeys
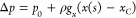
25where *p*_0_ is a background reference pressure. Combining eqs 20–25 yields a system of ordinary differential equations for the coordinates of the interface:
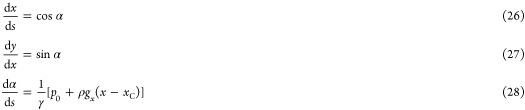
We impose boundary conditions in order to specify the position and the contact angles of the left and right edges of the droplet as follows:
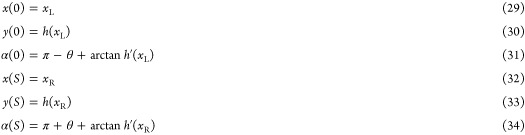
where *x*_L_ = *x*_C_ + *R* and *x*_R_ = *x*_C_ – *R* are the *x*-coordinates of the edges, and *R* and *S* are the droplet base radius and total arc length of the droplet surface (see Figure 1b) .

Equations 26–34 constitute a boundary-value problem for coordinates of the interface. For a given choice of the position of the droplet, *x*_C_; base radius, *R*; and contact angle, θ, one has three unknown parameters, namely, the background pressure, *p*_0_; the force density, ρ*g*_*x*_; and the total arc length, *S*. Once these are determined, one can compute the droplet cross-sectional area, *A*, and the response force, *f*_r_. The stability of the droplet to the displacement is then determined by the sign of *f*_r_ as discussed above.

To solve the boundary-value problem we used a fourth-order collocation algorithm similar to the one reported in ref (15) implemented in Python using the solve_bvp function from the scipy package. The unknown parameters are found using a root-finding Newton–Raphson algorithm, which minimizes the error accumulated from the approximation of the boundary conditions and the solution of the differential equations.

## III Results

**Figure 3:**
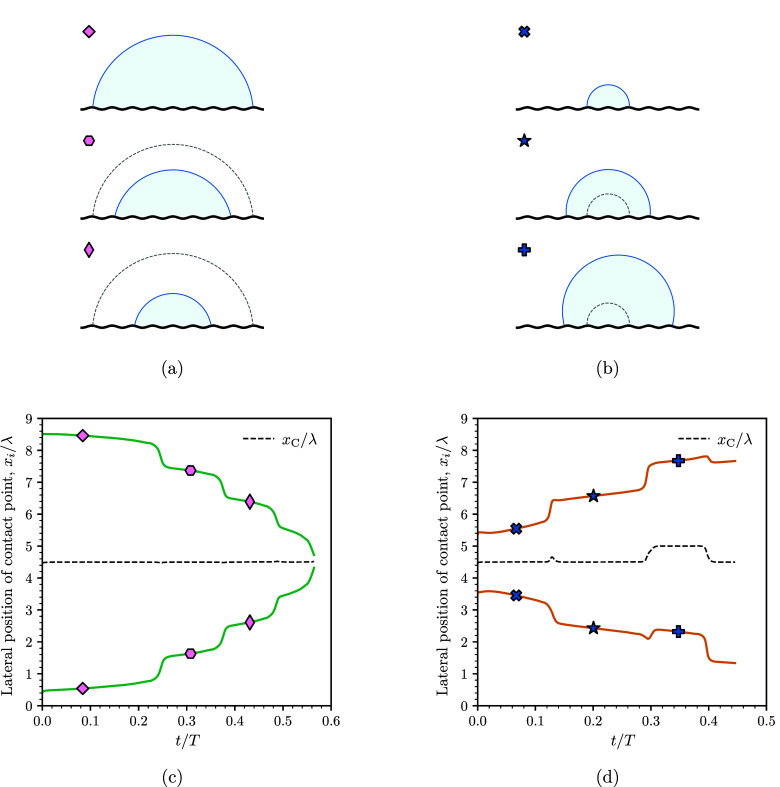
Snap events during a decrease and an increase in droplet size. (a and b) Simulation snapshots for growing (a) and shrinking (b) droplets initially centered on a peak of the surface (dashed profile). (c and d) Evolution of the contact points, *x*_L_ and *x*_R_ (solid lines), and droplet center, *x*_C_ (dashed line). The markers correspond to the snapshots shown in panels a and b.

We start by comparing the time evolution of growing and shrinking droplets in contact with the sinusoidal surface. We fix the contact angle to θ = 90°, the standard deviation to σ_θ_ = 0.13° and the amplitude of the pattern to *a* = 0.0527λ. Simulation snapshots for shrinking and growing droplets are shown in Figure 3a,b, respectively. The corresponding instantaneous positions of the contact points, *x*_L_(*t*) and *x*_R_(*t*), are shown in Figure 3c,d. Initially, *x*_L_ and *x*_R_ vary slowly. This slow variation is interrupted at points where the edges of the drop undergo a sudden change in position. Such events, called snaps, have been reported previously for droplets evaporating on wavy smooth solid surfaces.^10^ We identify two types of snap events: symmetric snaps, where both contact points change simultaneously, so that the position of the center of the droplet remains constant; and asymmetric snaps, where the center of the droplet shifts laterally. For the parameters in these simulations, shrinking droplets only showed symmetric snaps, while growing droplets predominantly showed asymmetric snaps. We verified that setting the standard deviation to σ_θ_ = 0° eliminates the asymmetric snaps, and observed that both growing and shrinking droplets undergo symmetric snaps only. These observations suggest that, on the same surface, growing droplets are more likely to become unstable and undergo asymmetric snaps compared to shrinking droplets, and that this instability is triggered by the small heterogeneity of the contact angle

**Figure 4:**
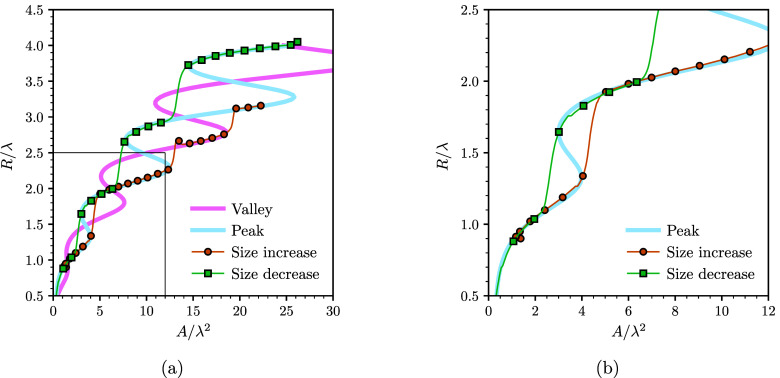
Droplet base radius vs cross-sectional area: (a) thick lines representing equilibrium configurations predicted by the Young–Laplace equation for a droplet centered on a peak or a valley of the sinusoidal surface and the thin lines with circle and square markers showing the simulation results of growing and shrinking droplets, respectively; (b) detail area of panel a. Snaps occur close to the lower and upper folds of the curve for growing and shrinking droplets, creating a hysteresis loop.

In two dimensions, there are two main kinds of stable equilibrium solutions of the Young–Laplace equation that determine the shape of a droplet in contact with a sinusoidal surface. These correspond to circular arcs that intersect the solid with the equilibrium contact angle, and are either aligned with a peak or a valley of the topography.^10^ For brevity, in the following we refer to these as peak and valley equilibrium solutions. As the droplet size varies, each of these solutions can be identified by tracking the base radius as a function of the cross-sectional area of the droplet, which we plot in Figure 4a. As shown in the figure, the base radius from the simulations slowly varies with the cross-sectional area following either the peak or valley equilibrium curves, thus indicating that the variation of the droplet size is a quasi-static process. This slow variation is interrupted during snaps, where the base radius varies much more rapidly, indicating that the droplet is out of equilibrium. For both growing and shrinking droplets, snaps occur close to the folds of the curves (see Figure 4b). For a growing droplet, the snaps occur close to the lower fold, while for a shrinking droplet they occur close to the upper fold. As a consequence, a droplet follows different paths during a size variation, creating a hysteresis loop. We note that the hysteresis loop arises from the different routes taken by the droplet during a size increase and decrease. This is similar to the hysteresis loop reported for simulations of droplets undergoing evaporation and condensation on sharp chemical patterns.^16^

**Figure 5:**
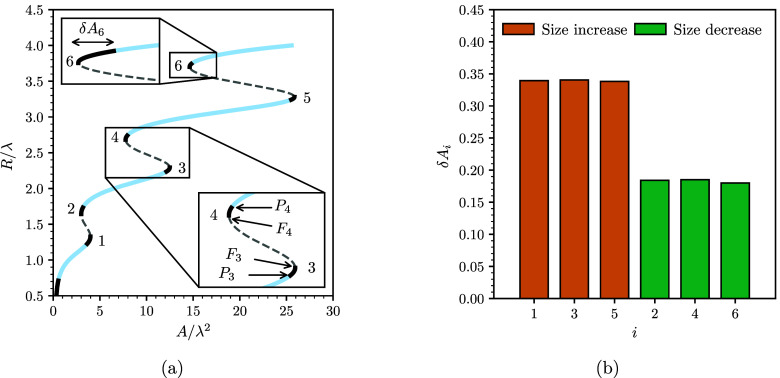
Droplet stability. (a) Stable and unstable configurations of a peak centered droplet on the sinusoidal surface. The blue and dashed branches correspond to stable and unstable configurations, respectively. The solid black lines indicate unstable configurations to lateral displacements, and are delimited by fold (*F*_*i*_) and pitchfork (*P*_*i*_) bifurcation points (see points *F*_3_, *P*_3_, *F*_4_, and *P*_4_ in the inset). The separation between adjacent fold and pitchfork bifurcation points is quantified in terms of the band widths, δ*A*_*i*_, for growing droplets (odd numbers) and shrinking droplets (even numbers). (b) Comparison of the separation between fold and pitchfork bifurcation points between growing and shrinking droplets. The index *i* corresponds to the band numbers in panel a.

To understand why growing droplets are more likely to undergo asymmetric snaps, we conducted a stability analysis of the peak and valley equilibrium solutions using the sharp interface formulation presented in Section II.B. Figure 5a shows the results of the stability analysis for the peak equilibrium curve (results for a valley curve are qualitatively the same). The blue segments in the curve correspond to configurations that are stable upon a lateral displacement, while the black thick lines correspond to unstable configurations. The dashed lines are fully unstable configuration, i.e., unstable to both changes in droplet radius and position, as reported in ref (10) and were not observed in the simulations.

Symmetric snaps occur at the folds of the *R*(*A*) curve, where the droplet transitions from the last equilibrium solution at the fold to a new equilibrium solution with the same cross-sectional area. Such snaps are caused by a saddle-node bifurcation.^10^ The new equilibrium solution is located in the same curve, and has either a higher or a lower base radius, depending on which fold the snap occurs. For instance, for a growing droplet, for which snaps occur on the lower folds, the new solution has a higher base radius. In contrast, asymmetric snaps are not restricted to a single point on the curve, but can happen over a range of droplet sizes determined by the unstable bands. Such snaps arise due to a pitchfork bifurcation, where two unstable asymmetric solutions merge with the symmetric peak (or valley) curve.^10^ As a result, the droplet shifts laterally to the adjacent equilibrium curve.

We now discuss the likelihood of the droplet to undergo an asymmetric snap by examining the unstable bands in more detail. Let us number each band in order of growing base radius as shown in Figure 5a. Each band is delimited by a fold bifurcation point, *F*_*i*_, and a pitchfork bifurcation point, *P*_*i*_. The separation distances between these points, δ*A*_*i*_ = |*A*(*P*_*i*_) – *A*(*F*_*i*_)| (see inset in Figure 5a), determine the range of areas over which the droplet is unstable to lateral perturbations, and are thus a measure of the likelihood observing an asymmetric snap. As shown in Figure 5b, the fold–pitchfork point separation is larger for growing droplets (*i* = 1, 3, 5) than for shrinking droplets (*i* = 2, 4, 6). This result indicates that the likelihood of triggering asymmetric snaps for growing droplets is also higher, which is captured in the numerical simulations.

**Figure 6:**
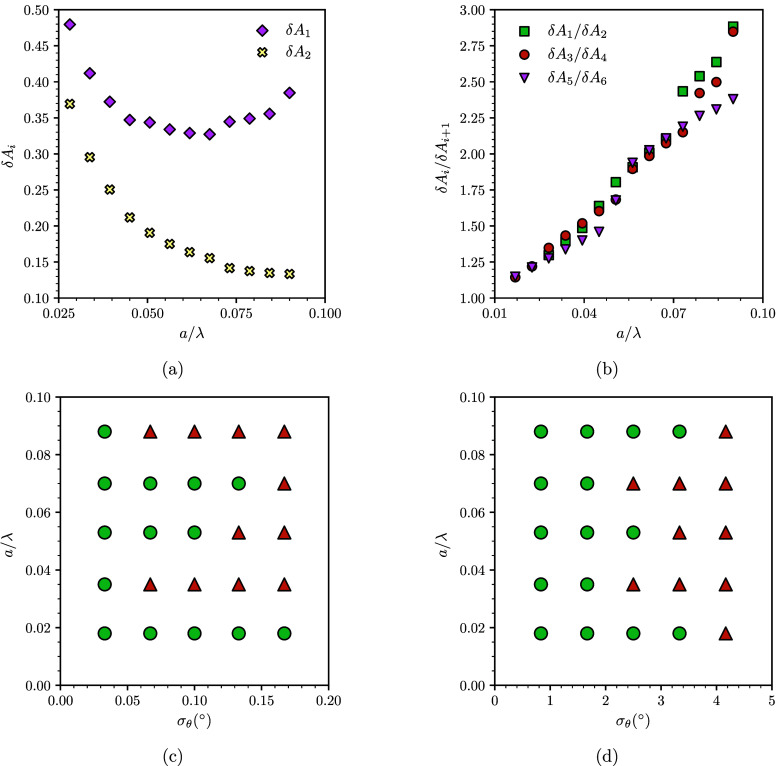
Effect of the amplitude of the surface, *a*, and the standard deviation of the contact angle, σ_θ_, on the competition between symmetric and asymmetric snaps. (a) Variation of fold–pitchfork point separation for paths of growing (δ*A*_1_) and shrinking (δ*A*_2_) droplets vs amplitude of the pattern. (b) Ratio of fold–pitchfork point separation between paths of growing and shrinking droplets vs amplitude of the pattern. (c and d) Phase maps of symmetric and asymmetric snaps for growing droplets (c) and shrinking droplets (d). Green circles represent results where all snaps are symmetric. Red triangles represent results where at least one snap is asymmetric. The strength of the standard deviation of the contact angle needed to trigger asymmetric snaps is significantly larger for shrinking droplets.

To explore this effect in more depth, we measured the δ*A*_*i*_ for different values of the amplitude of the sinusoidal pattern, a. As shown in Figure 6a, δ*A*_2_ (corresponding to an upper fold and to the path taken by a shrinking droplet) decreases with increasing a, suggesting that, for shrinking droplets, asymmetric snaps become less likely as a increases. On the other hand, δ*A*_1_ (lower fold, growing droplet) has a nonmonotonic dependence on *a*, initially decreasing before increasing again at higher amplitudes. In general, the δ*A*_*i*_ are always larger on lower folds, and their relative size compared to the closest upper fold increases with amplitude. These results suggest that, for shrinking droplets, asymmetric snaps become less likely on surfaces of larger amplitude (see Figure 6b). On the other hand, for growing droplets we expect that asymmetric snaps become less frequent by increasing the amplitude, before becoming more frequent again as shown on Figure 6a. To verify this prediction, we carried out simulations of growing and shrinking droplets at different values of *a* and σ_θ_. For each combination of parameters, we performed two independent simulations, each using a different seed for the noise in the contact angle, and determined whether the droplet undergoes at least one asymmetric snap.

Panels c and d of Figure 6 show the resulting phase maps, where circles denote simulations where only symmetric snaps were observed and triangles to simulations where at least one asymmetric snap was observed. Each point corresponds to 4–6 snaps, giving a total of 100–150 snap events across the whole sweep in *a* and σ_θ_. As expected, for growing droplets we observe asymmetric snaps at low and high amplitudes (Figure 6c), with most snaps at high amplitudes occurring close to the first unstable band of the *R*(*A*) curve (for which δ*A*_1_ is nonmonotonic). This result contrasts with the behavior of shrinking droplets, for which increasing the amplitude always leads to symmetric snaps (Figure 6d), as expected from the monotonic decrease of δ*A*_2_ with increasing a.

**Figure 7:**
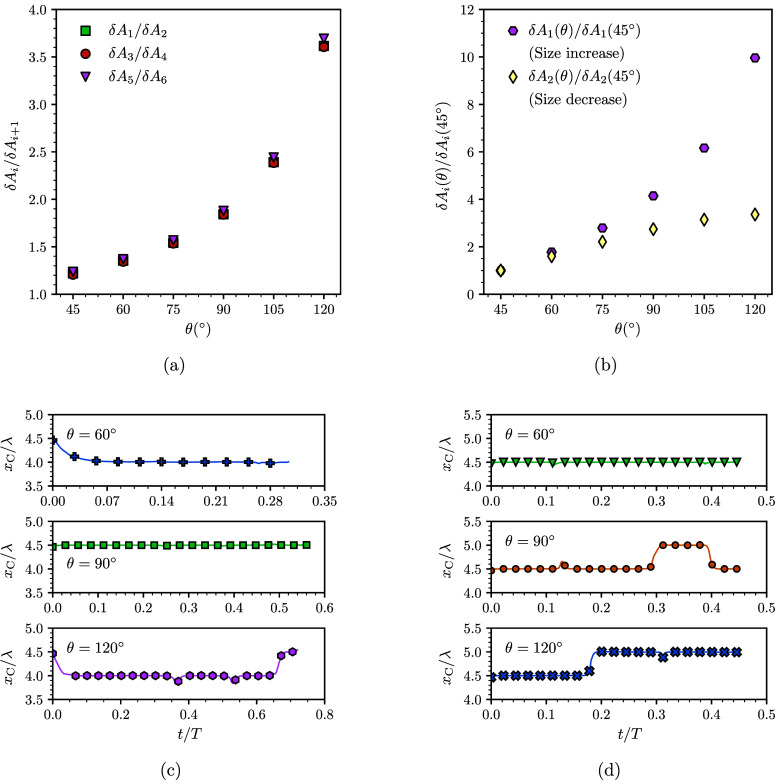
Effect of the contact angle of the solid surface on the competition between symmetric and asymmetric snaps. (a) Ratio of fold–pitchfork point separation between paths of growing and shrinking droplets vs the equilibrium contact angle, θ. (b) Variation of fold–pitchfork point separation for paths of growing (δ*A*_2_) and shrinking (δ*A*_1_) droplets vs θ. (c and d) Evolution of the center position of a droplet of equilibrium contact angles θ = 60°, 90°, and 120°, during a decrease (c) and an increase (d) in size.

The phase maps of Figure 6c,d also illustrate the sharp contrast in sensitivity of growing vs shrinking droplets to undergo asymmetric snaps. While it is possible to trigger asymmetric snaps for shrinking droplets by increasing the standard deviation in the distribution of the contact angle, the typical values needed are at least 1 order of magnitude higher than for growing droplets. This is consistent with the overall larger unstable bands for growing droplets vs shrinking droplets reported in Figure 5a. We now turn our attention to the effect of the equilibrium contact angle of the surface on the stability of the droplet. In Figure 7a we plot the ratio of the δ*A*_*i*_ between growing and shrinking droplets for different contact angles. As before, the lower bands (on the path of growing droplets) are always larger than the upper bands and the ratio between the two increases with the equilibrium contact angle. Figure 7b shows the variation of δ*A*_1_ and δ*A*_2_ with θ, relative to θ = 45°. While both δ*A*_1_ and δ*A*_2_ increase with θ, δ*A*_1_ does so more rapidly. This trend suggests that it is more likely to observe asymmetric snaps for a growing droplet on a surface with higher contact angle. Figure 7 shows simulation results for droplets on surfaces where θ = 60°, 90°, and 120°. For shrinking droplets (Figure 7c), the center of the droplet remains at the same position for θ = 60° and θ = 90°, indicating symmetric snaps (the variation at early times for θ = 60° and θ = 120° corresponds to the relaxation of the droplet from the initial condition). For a higher contact angle, θ = 120°, the droplet undergoes at least one asymmetric snap. For a growing droplet, asymmetric snaps are observed for both θ = 90° and θ = 120° and it is only at low contact angles that symmetric snaps are observed. Therefore, under the same conditions, growing droplets are more likely to undergo asymmetric snaps, and this likelihood increases with increasing contact angle, in broad agreement with the prediction of the stability analysis.

## IV Conclusions

We have investigated the stability of growing and shrinking droplets in contact with a smooth wavy solid surface and focused on understanding the competition between symmetric and asymmetric snaps that arise as the size of the droplet varies. Using hydrodynamic simulations we have identified that asymmetric snaps are more likely to occur for droplets undergoing a growth in size than for droplets decreasing in size. Asymmetric snaps are more likely for growing droplets on surface patterns of large amplitude, but less likely for droplets shrinking in size on the same pattern. We have also studied the effect of the contact angle of the surface, and found that asymmetric snaps become more likely on surfaces of high equilibrium contact angle. We have studied the stability of the droplets around equilibrium states by solving the Young–Laplace equation. We have rationalized the results from the numerical simulations by quantifying the distance between fold and pitchfork bifurcation points, which determines the range of droplet sizes that are unstable to lateral perturbations, and have found a clear correlation between these ranges and the observed occurrence of asymmetric snaps in the simulations. Therefore, the competition between asymmetric and symmetric snaps reported here arises from the interplay between fold and pitchfork bifurcation points.

Our work highlights the crucial role of the structure of a solid surface in the processes of growth or decrease of size of a droplet, and provides a framework to design surfaces that achieve better manipulation and control of droplets during a variation of droplet size. Here we have focused on two-dimensional droplets on a sinusoidal topography as a model system but our approach can be generalized to three dimensions^10^ and to model the effect of a smooth chemical pattern.^11,17^ Our modeling approach can also be used to study the probability of observing different types of snaps on surfaces of controlled topography. Experimentally, smooth surface topographies of designed shape can be achieved using slippery liquid–infused porous surfaces,^18−21^ where a lubricant layer is added to an underlying surface, or via liquid-like surfaces, where a smooth polymer coating is covalently attached to the solid.^22−25^ We hope that our results guide the rational design of these surfaces, which can help improve applications that involve droplets subject to a change in size in contact with solids, such as printing, fog harvesting, self-cleaning and drying.

Finally, our results are relevant in other systems where pitchfork and bifurcation points occur in pairs. For instance, fold and pitchfork bifurcations are known to underpin the snap-through instabilities observed in elastic beams and sheets^26^ which have relevance in microactuators^27^ and soft robotics.^28^

## Supporting Information Available

Lattice–Boltzmann simulation method and simulation parameters; link for open-source code for boundary-value problem (PDF)
