# Supplementary material for: Competing Bifurcations Determine Symmetry Breaking During Droplet Snaps on Smooth Patterned Surfaces
Source: Langmuir. 2024 Nov 6;40(46):24387–96. doi: 10.1021/acs.langmuir.4c02908 (PMC11580171; doi:10.1021/acs.langmuir.4c02908)
Supplement: Supplementary file 1 — la4c02908_si_001.pdf [file la4c02908_si_001.pdf]

Competing Bifurcations Determine Symmetry Breaking During Droplet Snaps on Smooth Patterned Surfaces
Supplementary Information

Lucile Bisquert,[1] Élfego Ruiz-Gutiérrez,[2] Marc Pradas,[3] and Rodrigo Ledesma-Aguilar[1,*]

[1]*Institute for Multiscale Thermofluids, School of Engineering, University of Edinburgh, The King's Buildings, Mayfield Road, Edinburgh EH9 3FB, United Kingdom*

[2]*School of Engineering, Newcastle University, Claremont Road, Newcastle upon Tyne NE1 7RU, United Kingdom*

[3]*School of Mathematics and Statistics, The Open University, Milton Keynes, MK7 6AA, United Kingdom*

[*] rodrigo.ledesma@ed.ac.uk

CONTENTS

Lattice-Boltzmann Simulation method S2

Lattice-Boltzmann Simulation Parameters S5

Boundary Value Problem: Open source code S6

References S6

LATTICE-BOLTZMANN SIMULATION METHOD

To integrate the Navier-Stokes and Cahn-Hilliard equations we use the Lattice-Boltzmann Method (LBM) [1–3]. Here, we provide a brief summary of the method with its specifications. However, the reader is referred to Krüger, et al. [4] for a more detailed discussion on the method.

The continuity and Navier-Stokes equations are solved by means of the Boltzmann equation discretised in time, space and peculiar velocity in the following way,

$$f_q(\vec{r}+\vec{c}_q\Delta t, t+\Delta t) - f_q(\vec{r},t) = -\frac{1}{\tau_f}\left(f_q - f_q^e\right) + \Delta t F_q, \tag{S.1}$$

where $f_q(\vec{r},t)$ are the distribution functions, where the index q denotes a particular lattice velocity vector, $\vec{c}_q$. Here, we employ a D2Q9 model [5], which consists of a 3×3 square lattice with 9 velocity vectors. The density and velocity fields are defined by the moments of the distribution function, $\rho(\vec{r},t) = \sum_q f_q$ and $\vec{u}(\vec{r},t) = (\sum_q \vec{c}_q f_q)/\rho$, respectively.

The left-hand side of Eq. (S.1) corresponds to a propagation step, where the distribution functions are advected along the links of the lattice over a time step Δt. The first term on the right-hand side of the equation corresponds to a collision step. Here we consider the BGK approximation from Kinetic Theory [6, 7], where τ_f corresponds to a relaxation time that maps to the kinematic viscosity via $\tau_f = \nu c_s^{-2} + 1/2$. In this step, the distribution functions relax towards an equilibrium distribution function, f_q^e, given by

$$f_q^e(\rho,\vec{u}) = w_q\rho\left(1 + \frac{\vec{u}\cdot\vec{c}_q}{c_s^2} + \frac{\vec{u}\vec{u} : (\vec{c}_q\vec{c}_q - \mathbf{I}c_s^2)}{2c_s^4}\right). \tag{S.2}$$

where $w_q = 4^{1-|\vec{c}_q|^2}/9$ correspond to quadrature weights. The last term in Eq. (S.1) is used to introduce the force-density term $-\phi\nabla\mu$ in the Navier-Stokes equations, which we implement

employing Guo's forcing scheme [8],

$$F_q = -\left(1 - \frac{\Delta t}{2\tau}\right) w_q \left[\frac{\vec{c}_q - \vec{u}}{c_s^2} + \frac{(\vec{c}_q \cdot \vec{u})\vec{c}_q}{c_s^4}\right] \cdot (\phi \vec{\nabla} \mu). \tag{S.3}$$

The Cahn-Hilliard equation is solved by a second lattice-Boltzmann equation that is integrated simultaneously,

$$g(\vec{r} + \Delta t \vec{c}_q, t + \Delta t) - g(\vec{r}, t) = -\frac{1}{\tau_g}(g_q - g_q^e), \tag{S.4}$$

where $\phi = \sum_q g_q$ and the equilibrium distribution is defined by

$$g_q^e(\phi, \vec{u}, \mu) = \phi \delta_{0q} + \phi w_q \left[\frac{\vec{u} \cdot \vec{c}_q}{c_s^2} + \frac{\vec{u}\vec{u} : (\vec{c}_q \vec{c}_q - \mathbf{I} c_s^2)}{2 c_s^4}\right] + \frac{2 w_q M \mu}{c_s^2}, \tag{S.5}$$

where δ_{rq} is the Kronecker delta. Here, we use $\tau_g = 1$, which gives stability and robustness to the numerical method without loss in accuracy.

The boundary conditions are implemented as follows. We use an interpolated bounce-back algorithm for the solid boundary [9],

$$f_{\bar{q}}(\vec{r}, t + \Delta t) = \frac{\delta_q}{1 + \delta_q} f_q^{\star}(\vec{r}, t) + \frac{1 - \delta_q}{1 + \delta_q} f_q^{\star}(\vec{r} - \vec{c}_q \Delta t, t) + \frac{\delta_q}{1 + \delta_q} f_{\bar{q}}^{\star}(\vec{r}, t), \tag{S.6}$$

for the unknown particle population $f_{\bar{q}}$ travelling in the opposite direction to the solid surface, that is, $\bar{q}$ is such that $\vec{c}_{\bar{q}} + \vec{c}_q = 0$. The distance fraction, δ_q, is such that $\vec{r}_{\mathrm{b}} = \vec{r} + \delta_q \vec{c}_q$, where $\vec{r}_{\mathrm{b}}$ is the position of the solid surface. In Eq. (S.6), $f_q^{\star}$ corresponds to the pre-streaming particle population, i.e., before updating the populations to $t + \Delta t$. This ensures that the velocity satisfies the boundary condition, as in Eq. (12) of the main text, and conserves mass density, as in Eq. 13 of the main text, with an accurate representation for the shape of the boundary. Similarly,

$$g_{\bar{q}}(\vec{r}, t + \Delta t) = \frac{\delta_q}{1 + \delta_q} g_q^{\star}(\vec{r}, t) + \frac{1 - \delta_q}{1 + \delta_q} g_q^{\star}(\vec{r} - \vec{c}_q \Delta t, t) + \frac{\delta_q}{1 + \delta_q} g_{\bar{q}}^{\star}(\vec{r}, t), \tag{S.7}$$

ensures that Eqs. (9) and (11) from the main text are satisfied.

At the open boundary, we implement an anti-bounceback algorithm [10],

$$f_{\bar{q}}(\vec{r}, t + \Delta t) = \frac{1}{2}\left[f_{\bar{q}}^e(\rho_H, \vec{u}_H) + f_q^e(\rho_H, \vec{u}_H)\right], \tag{S.8}$$

which satisfies Eqs. (16) and (17) from the main text by extrapolating the velocity at the boundary,

$\vec{u}_H = \vec{u}(\vec{r})$, which is assumed to be at $\vec{r} + \vec{c}_q \Delta t/2$. Similarly,

$$g_{\bar{q}}(\vec{r}, t + \Delta t) = \frac{1}{2}\left[g^e_{\bar{q}}(\phi_H, \vec{u}_H, \mu_H) + g^e_q(\phi_H, \vec{u}_H, \mu_H)\right], \tag{S.9}$$

which satisfies the boundary conditions in Eqs. (14) and (15) from the main text.

The calculation of the gradient and Laplacian via finite differences at near-boundary nodes, such as Eqs. (9), (11), (14) and (15) of the main text, require special attention. For this, we follow Ref. [11] by carrying out a Taylor series expansion [12] of the cut-links, $q \in \Gamma_c$, as follows. For a Dirichlet boundary condition this is

$$\delta_q \vec{c}_q \cdot \nabla\phi(\vec{r}) + \frac{1}{2}\delta_q^2 \vec{c}_q \vec{c}_q : \nabla\nabla\phi(\vec{r}) = \mathcal{D} - \phi(\vec{r}) \tag{S.10}$$

where $\mathcal{D}$ represents the constant of the Dirichlet boundary value. Then, for Neumann boundary conditions, the expansion reads

$$\hat{n}_q \cdot \nabla\phi(\vec{r}) + \delta_q \hat{n}_q \vec{c}_q : \nabla\nabla\phi(\vec{r}) = \mathcal{N}, \tag{S.11}$$

where $\mathcal{N}$ is the Neumann constant at the boundary and $\hat{n}_q$ is the local unitary normal vector to the surface. For the rest of the field values, $q \notin \Gamma_c$, the Taylor series around $\vec{r}$ this is given by,

$$\vec{c}_q \cdot \nabla\phi(\vec{r}) + \frac{1}{2}\vec{c}_q \vec{c}_q : \nabla\nabla\phi(\vec{r}) = \phi(\vec{r} + \vec{c}_q) - \phi(\vec{r}). \tag{S.12}$$

In these equations, $\nabla\phi(\vec{r})$, the gradient vector, and $\nabla\nabla\phi(\vec{r})$, the Hessian matrix, are unknown variables. In two-dimensional space, the gradient vector (2) and the Hessian matrix (3) together consist of 5 independent components.

Eqs. (S.10) or (S.11) together with (S.12) provide $Q - 1 = 8$ equations, therefore, the linear system of equations is over-specified. To address this problem, we introduce the following method to solve the system.

Let us begin by levelling every instance of Eq. (S.11) to the same units of Eq. (S.12) by multiplying $\delta_q |\vec{c}_q|^2 / \hat{n}_q \cdot \vec{c}_q$. Then, we convert the system into matrix form:

$$\mathbf{G}\vec{\Lambda} = \vec{\Phi}, \tag{S.13}$$

where

$$\vec{\Lambda} := \left(\partial_x\phi,\ \partial_y\phi,\ \partial_x^2\phi,\ \partial_y^2\phi,\ \partial_x\partial_y\phi\right)^T(\vec{r}) \tag{S.14}$$

is the vector containing the unknown elements of the gradient vector and the Hessian matrix at $\vec{r}$. The vector $\vec{\Phi}$ is composed of known field values and boundary conditions, represented as:

$$\Phi_q := \begin{cases} \mathcal{D} - \phi(\vec{r}) & \text{if } q \in \Gamma_c \text{ and a Dirichlet BC,} \\ \delta_q \mathcal{N} |\vec{c}_q|^2 / \hat{n}_q \cdot \vec{c}_q & \text{if } q \in \Gamma_c \text{ and a Neumann BC,} \\ \phi(\vec{r} + \vec{c}_q) - \phi(\vec{r}) & \text{otherwise.} \end{cases} \tag{S.15}$$

and $\mathbf{G}$ is a 8×5 coefficient matrix representing the local structure of the field and its boundaries that come from the left-hand side of Eqs. (S.10)–(S.12).

The pseudo-inverse algorithm estimates the solution $\vec{\Lambda}$ using $\mathbf{G}^{-1}$, calculated as:

$$\mathbf{G}^{-1} := (\mathbf{W}\mathbf{G})^{-1}\mathbf{W}. \tag{S.16}$$

Here, $\mathbf{W}$ is a 5×8 projection matrix transforming $\mathbf{G}$ into a square matrix 5×5 matrix, defined by:

$$W_q := \frac{w_q}{\delta_q c_s^2}\left(\frac{c_{xq}}{\delta_q},\ \frac{c_{yq}}{\delta_q},\ \frac{c_{xq}^2}{c_s^2},\ \frac{c_{yq}^2}{c_s^2},\ \frac{c_{xq}c_{yq}}{c_s^2}\right)^T. \tag{S.17}$$

In Eq. (S.17), we have generalised $\delta_q = 1$ for $q \notin \Gamma_c$. The stencil for approximating the 5 derivatives would only require 5 linearly independent points to make the system of equations have a unique solution. This would require choosing only 5 out of the 8 points in the neighbourhood [12]. However, the choice of those five points is not unique, and the solution may be biased. A method to alleviate this bias is to use the 5x8 projection matrix as expressed in Eq. (S.16), which assigns different weights to the points in the neighbourhood. Following Ref. [13], we use the projection matrix in Eq. (S.17), which preserves isotropy and has been validated in Ref. [11].

Since the matrix $\mathbf{G}$ represents the static lattice and boundary structure, the pseudo-inverse algorithm in Eq. (S.16) is implemented during the initialization of the simulation. Thus, it is no more computationally intensive than the typical application of a finite-differences stencil.

LATTICE-BOLTZMANN SIMULATION PARAMETERS

Simulation parameters are reported in Tables I and II.

(L_x, L_y)	λ	ℓ	M	γ	ν_+	ν_-	x_{C}	$\vec{u}(\vec{r})$	$\rho(\vec{r})$
$(128, 128)$	$L_x/9$	1.4	18	1×10^{-4}	0.083	0.0083	$0.5L_x$	$\vec{0}$	1

TABLE I: Model parameters. These parameters are fixed throughout all the simulations. (L_x, L_y) corresponds to the dimension of the domain, λ to the wavelength of the sinusoidal surface, ℓ to the interface thickness, M to the mobility and γ to the surface tension. The viscosity of the droplet is ν_+ and the viscosity of the surrounding gas phase is ν_-. The initial position of the centre of the droplet is x_{C}. The velocity field is $\vec{u}(\vec{r})$ and density field is $\rho(\vec{r})$.

Case	R	ϕ_{H}	θ	σ_θ	a
Growing	$0.1L_x$	-0.7	$45° \leq \theta \leq 120°$	$0° \leq \sigma_\theta \leq 5°$	$0.018\lambda \leq a \leq 0.088\lambda$
Shrinking	$0.45L_x$	-1.3	$45° \leq \theta \leq 120°$	$0° \leq \sigma_\theta \leq 5°$	$0.018\lambda \leq a \leq 0.088\lambda$

TABLE II: Simulation parameters for growing and shrinking droplets. R corresponds to the base radius of the droplet, ϕ_{H} to the phase field at the top boundary, θ to the equilibrium contact angle, σ_θ to the standard deviation of the contact angle distribution, and a to the amplitude of the sinusoidal surface.

BOUNDARY VALUE PROBLEM: OPEN SOURCE CODE

The Python code used to solve the boundary-value problem is available as an open-source code. See Ref. [14].

REFERENCES

[1] Briant, A. J.; Yeomans, J. M. Lattice Boltzmann simulations of contact line motion. II. Binary fluids. *Phys Rev E* **2004**, *69*, 031603.

[2] Ledesma-Aguilar, R.; Vella, D.; Yeomans, J. M. Lattice-Boltzmann simulations of droplet evaporation. *Soft Matter* **2014**, *10*, 8267–8275.

[3] Wells, G.; Ruiz-Gutiérrez, É.; Le Lirzin, Y.; Nourry, A.; Orme, B.; Pradas, M.; Ledesma-Aguilar, R. Snap evaporation of droplets on smooth topographies. *Nat Commun* **2018**, *9*, 1380.

[4] Krüger, T.; Kusumaatmaja, H.; Kuzmin, A.; Shardt, O.; Silva, G.; Viggen, E. M. *The Lattice Boltzmann Method: Principles and Practice*; Graduate Texts in Physics; Springer Cham, 2017.

[5] Shan, X.; Chen, H. Lattice Boltzmann model for simulating flows with multiple phases and components. *Phys Rev E* **1993**, *47*, 1815–1819.

[6] Bhatnagar, P. L.; Gross, E. P.; Krook, M. A Model for Collision Processes in Gases. I. Small Amplitude Processes in Charged and Neutral One-Component Systems. *Phys Rev* **1954**, *94*, 511–525.

[7] Lee, T.; Lin, C.-L. A stable discretization of the lattice Boltzmann equation for simulation of incompressible two-phase flows at high density ratio. *J Comput Phys* **2005**, *206*, 16–47.

[8] Guo, Z.; Zheng, C.; Shi, B. Discrete lattice effects on the forcing term in the lattice Boltzmann method. *Phys Rev E* **2002**, *65*, 046308.

[9] Yu, D.; Mei, R.; Luo, L.-S.; Shyy, W. Viscous flow computations with the method of lattice Boltzmann equation. *Prog Aerosp Sci* **2003**, *39*, 329–367.

[10] Ginzburg, I.; Verhaeghe, F.; d'Humieres, D. Two-relaxation-time lattice Boltzmann scheme: About parametrization, velocity, pressure and mixed boundary conditions. *Commun Comput Phys* **2008**, *3*, 427–478.

[11] Ruiz-Gutiérrez, E.; Ledesma-Aguilar, R. Lattice-Boltzmann simulations of the dynamics of liquid barrels. *J Phys Condens Matter* **2020**, *32*, 214007.

[12] Noye, B.; Arnold, R. Accurate finite difference approximations for the Neumann condition on a curved boundary. *Appl Math Model* **1990**, *14*, 2–13.

[13] Patra, M.; Karttunen, M. Stencils with isotropic discretization error for differential operators. *Numer Methods Partial Differ Equ* **2006**, *22*, 936–953.

[14] https://github.com/elfego/snapWavyForces/tree/master.
